# Real‐time detection of somatostatin release from single islets reveals hypersecretion in type 2 diabetes

**DOI:** 10.1111/apha.14268

**Published:** 2025-01-13

**Authors:** Mingyu Yang, Kousik Mandal, Moa Södergren, Özge Dumral, Lena Winroth, Anders Tengholm

**Affiliations:** ^1^ Department of Medical Cell Biology Uppsala University Uppsala Sweden

**Keywords:** cytoplasmic Ca^2+^, human islets, somatostatin secretion, SSTR2, type 2 diabetes, δ‐Cell

## Abstract

**Aim:**

Somatostatin from pancreatic δ‐cells is a paracrine regulator of insulin and glucagon secretion, but the release kinetics and whether secretion is altered in diabetes is unclear. This study aimed to improve understanding of somatostatin secretion by developing a tool for real‐time detection of somatostatin release from individual pancreatic islets.

**Methods:**

Reporter cells responding to somatostatin with cytoplasmic Ca^2+^ concentration ([Ca^2+^]_i_) changes were generated by co‐expressing somatostatin receptor SSTR2, the G‐protein Gα15 and a fluorescent Ca^2+^ sensor in HeLa cells.

**Results:**

Somatostatin induced dose‐dependent [Ca^2+^]_i_ increases in reporter cells with half‐maximal and maximal effects at 1.6 ± 0.4 and ~30 nM, respectively. Mouse and human islets induced reporter cell [Ca^2+^]_i_ elevations that were inhibited by the SSTR2 antagonist CYN154806. Depolarization of islets by high K^+^, K_ATP_ channel blockade or increasing the glucose concentration from 3 to 11 mM evoked concomitant elevations of [Ca^2+^]_i_ in islets and reporter cells. Exposure of islets to glucagon, GLP‐1 and ghrelin also triggered reporter cell [Ca^2+^]_i_ responses, whereas little effect was obtained by islet exposure to insulin, glutamate, GABA and urocortin‐3. Islets from type 2 diabetic human donors induced higher reporter cell [Ca^2+^]_i_ responses at 11 mM and after K^+^ depolarization compared with non‐diabetic islets, although fewer δ‐cells were identified by immunostaining.

**Conclusion:**

Type 2 diabetes is associated with hypersecretion of somatostatin, which has implications for paracrine regulation of insulin and glucagon secretion. The new reporter cell assay for real‐time detection of single‐islet somatostatin release holds promise for further studies of somatostatin secretion in islet physiology and pathophysiology.

## 1 INTRODUCTION

The peptide somatostatin is an important paracrine regulator of various physiological processes in the central nervous system, the gastrointestinal tract, and the pancreatic islets of Langerhans.^1^, ^2^ In islets, somatostatin is produced by δ‐cells, which constitute ~5% of the endocrine islet cell population.^3^ Despite their relatively low abundance, δ‐cells make close contacts with many insulin‐secreting β‐cells and glucagon‐secreting α‐cells via their extended processes,^4^, ^5^ and somatostatin thereby serves as a negative paracrine regulator of insulin and glucagon secretion.^6^, ^7^, ^8^, ^9^, ^10^ Somatostatin acts via G‐protein‐coupled receptors. Among five somatostatin receptor isoforms (SSTR1‐5), several have been detected in islets,^8^, ^11^, ^12^ with SSTR2 being the functionally dominant receptor in human α‐ and β‐cells.^13^ Like other somatostatin receptors, SSTR2 signals primarily via Gαi^1^ and somatostatin inhibits exocytosis in α‐ and β‐cells by multiple mechanisms, including lowering of cAMP,^14^ hyperpolarisation^15^ and activation of the protein phosphatase calcineurin.^16^, ^17^

The mechanisms regulating somatostatin secretion from δ‐cells are not well understood, but it has been demonstrated that glucose^18^, ^19^, ^20^ and various hormones and neurotransmitters, including GLP‐1,^21^ ghrelin,^22^, ^23^ urocortin‐3^24^ and glutamate,^25^ stimulate somatostatin release (see review^2^). In glucose‐stimulated islets, somatostatin is released in pulses synchronized with pulses of insulin, but in opposite phase to the glucagon pulses.^26^, ^27^ The synchronization between somatostatin and insulin seems to involve electrical coupling between β‐ and δ‐cells,^28^ whereas paracrine inhibitory effects likely explains the glucagon pulsatility.^29^

With its strong inhibitory effects on insulin and particularly glucagon secretion, somatostatin can be expected to influence whole body glucose homeostasis. Antagonizing the action of somatostatin has indeed been found to improve the glucagon response to hypoglycemia in diabetic rats.^30^, ^31^, ^32^ Moreover, in human diabetic patients, infusion of somatostatin was reported to improve glycemic control by reducing the hyperglucagonemia,^33^, ^34^, ^35^ but it is not known to which extent somatostatin secretion is altered in diabetes.

Studies of somatostatin secretion would benefit from improved methods for detection of the secreted peptide. Available immunoassays are specific and truly quantitative, but their sensitivity typically limits their application to large groups of islets and static incubations or perifusions with low temporal resolution. The aim of the present study was to develop a reporter cell assay for real‐time monitoring of somatostatin secretion from individual islets, allowing parallel recordings of islet signaling. The assay was used to characterize somatostatin release from mouse and human islets in response to glucose and various hormonal and neural stimuli and to investigate if somatostatin secretion is altered in islets of type 2 diabetic donors.

## 2 RESULTS

### 2.1 Generation of somatostatin reporter cells

**FIGURE 1:**
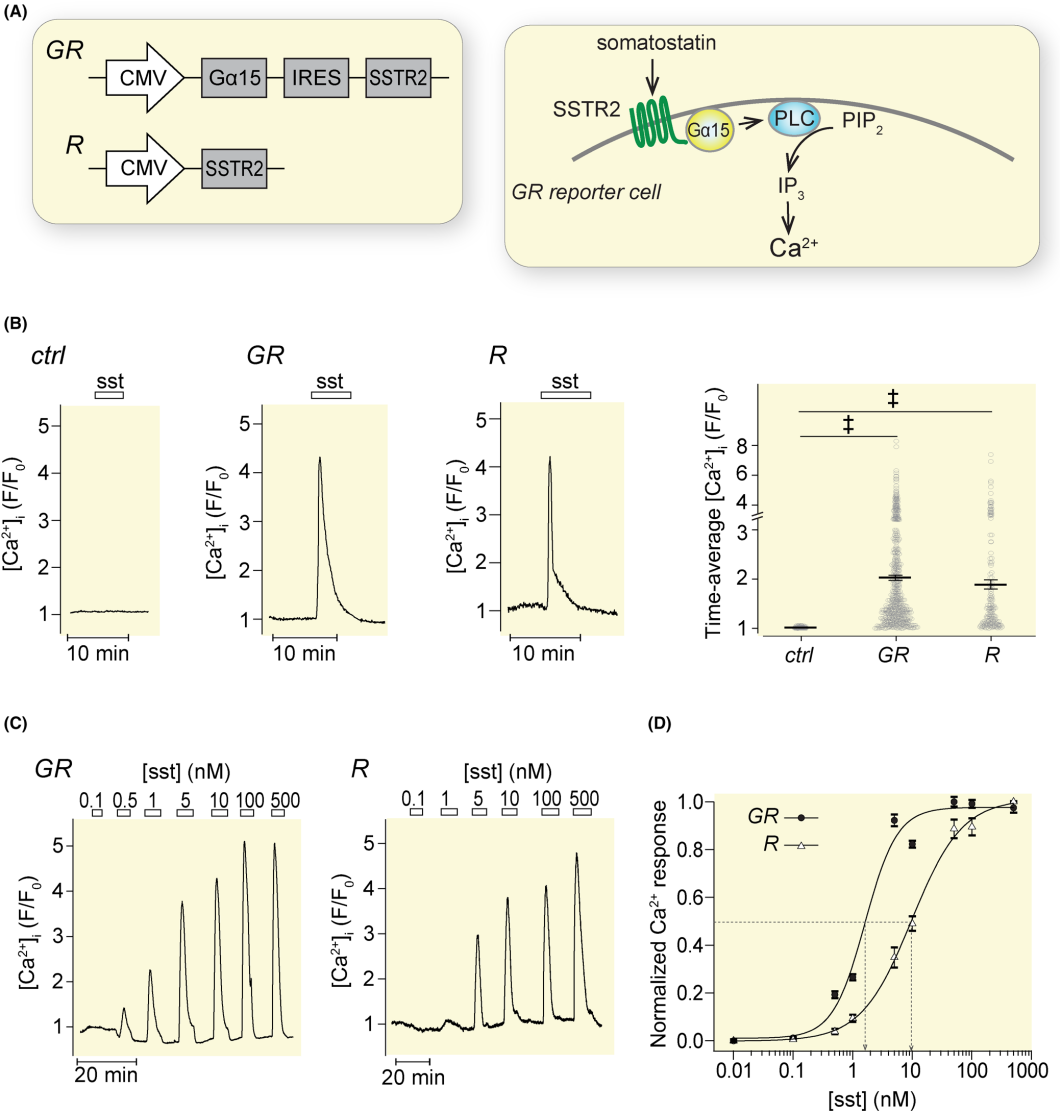
Generation of somatostatin reporter cells. (A) Plasmid constructs for SSTR2 expression with (GR) or without (R) Gα15 and schematic drawing of the somatostatin detection principle in a GR reporter cell. (B) Representative [Ca^2+^]_i_ recordings from single HeLa cells without SSTR2 expression (ctrl), with SSTR2 expression alone (R) or together with Gα15 (GR) during exposure to 100 nM somatostatin (sst). Quantification of the [Ca^2+^]_i_ responses are shown in scatter plots with mean ± SEM ‡*p* < 0.001 for difference from the pre‐stimulatory condition; Student's *t*‐test. Each dot represents the time‐averaged response from one cell. *n* = 129 cells from three experiments (ctrl), 190 cells from four experiments (GR cells) and 160 cells from three experiments (R‐cells). (C) [Ca^2+^]_i_ recordings from a single GR‐ and an R‐cell exposed to different concentrations of sst. (D) Mean ± SEM for the normalized [Ca^2+^]_i_ increases in GR (*n* = 135) and R (*n* = 133) cells (four experiments in each group). The data are fitted to a Hill equation and concentrations inducing half‐maximal fluorescence changes indicated.

To generate reporter cells for detection of somatostatin, HeLa cells were transfected either with an IRES construct containing SSTR2 and the Gq family protein Gα15 to link receptor activation to phospholipase C‐mediated Ca^2+^ signaling (GR‐cells; Figure 1A) or with SSTR2 alone (R‐cells). The cytoplasmic Ca^2+^ concentration ([Ca^2+^]_i_) was recorded with the Ca^2+^ reporter R‐GECO1 co‐transfected with the SSTR2 receptor constructs. There was no [Ca^2+^]_i_ response to somatostatin (100 nM) in HeLa cells transfected with Ca^2+^ reporter alone, whereas both GR‐ and R‐cells responded with a pronounced [Ca^2+^]_i_ peak followed by decline to baseline within 5–10 min (Figure 1B). Somatostatin triggered [Ca^2+^]_i_ increases in all cells transfected with SSTR2. The time‐averaged responses are shown in Figure 1B.

The sensitivity to somatostatin was assessed by addition of increasing concentrations of the hormone from 0.1 to 500 nM. GR‐cells showed dose‐dependent [Ca^2+^]_i_ elevations with half‐maximal and maximal effects at 1.6 ± 0.4 nM and ~30 nM, respectively (Figure 1C,D). R‐cells were less sensitive with half‐maximal and maximal concentrations at 9.8 ± 0.9 nM and ~500 nM, respectively, and unless otherwise stated, the subsequent experiments were therefore performed with GR‐cells.

### 2.2 Reporter cell detection of somatostatin release from pancreatic islets

**FIGURE 2:**
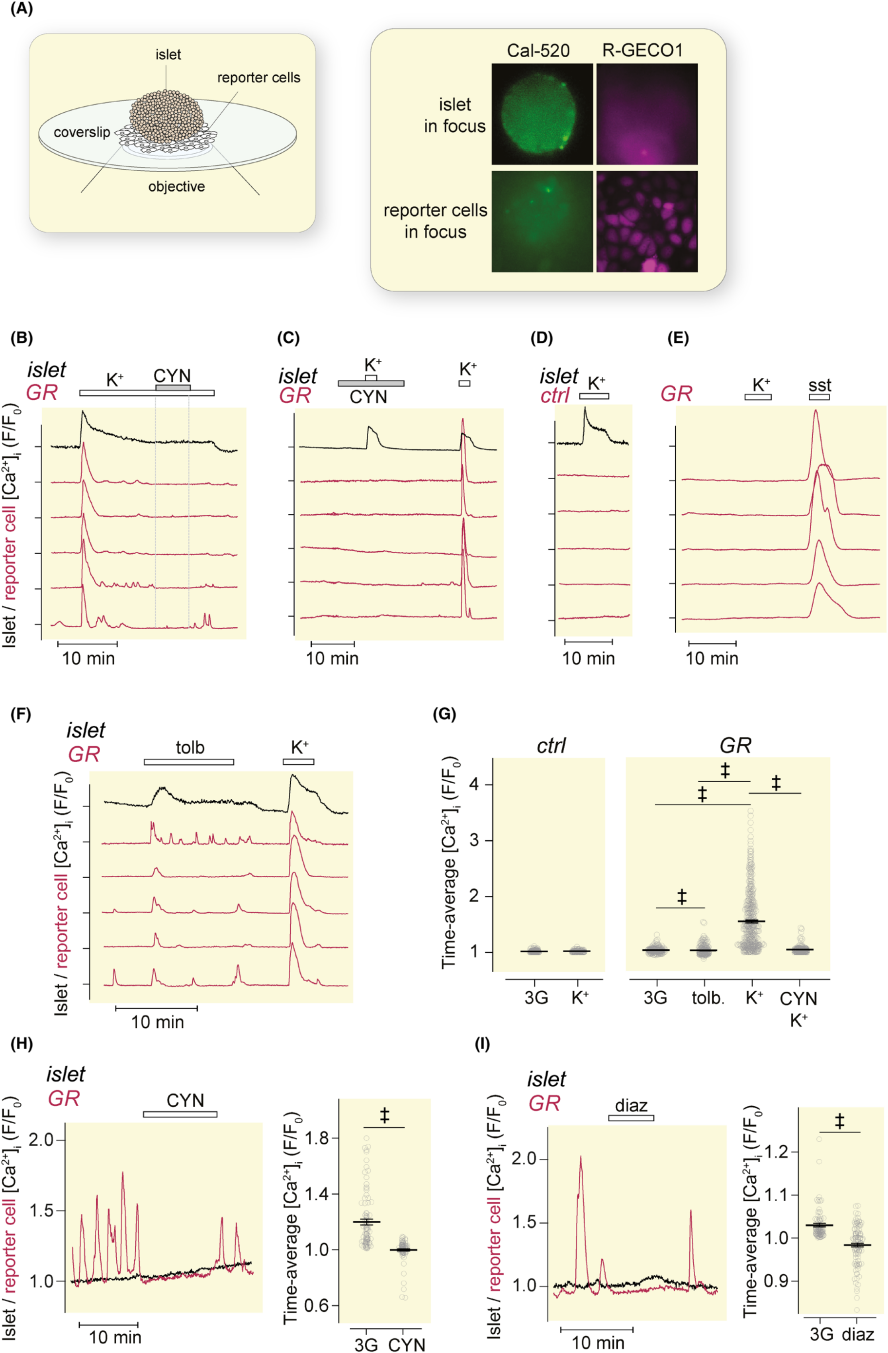
Detection of somatostatin from pancreatic islets. (A) Experimental setup for detection of somatostatin release from a pancreatic islet with reporter cells. The images show fluorescence from the two spectrally distinct Ca^2+^ indicators with either the Cal‐520‐loaded islet or the R‐GECO1‐expressing reporter cells in focus. (B, C) [Ca^2+^]_i_ recordings from a mouse islet (black trace) and adjacent GR‐cells (red traces) during stimulation with 30 mM K^+^ and exposure to 200 nM CYN154806. (D) [Ca^2+^]_i_ recordings from a mouse islet and control reporter cells during stimulation with 30 mM K^+^. (E) [Ca^2+^]_i_ recordings from GR‐cells in the absence of islets during exposure to 30 mM K^+^ and 100 nM somatostatin. (F) [Ca^2+^]_i_ recordings from a mouse islet and GR‐cells during stimulation with 100 μM tolbutamide and 30 mM K^+^. (G) Time‐averaged [Ca^2+^]_i_ responses with mean ± SEM for experiments as in B, D, and F. 3G refers to basal conditions with buffer containing 3 mM glucose. ‡*p* < 0.001 for the difference (Student's *t*‐test). *n* = 199 (ctrl); 200 (GR, K^+^ and CYN) and 114 (tolb and K^+^) cells from three experiments in each group. (H, I) [Ca^2+^]_i_ recordings from GR‐cells with spontaneous [Ca^2+^]_i_ increases (red) and mouse islets (black) under basal conditions with 3 mM glucose and after application of 200 nM CYN154806 (H, *n* = 107 cells, three experiments) or 250 μM diazoxide (I, *n* = 93 cells, three experiments). Time‐averaged [Ca^2+^]_i_ responses with mean ± SEM ‡*p* < 0.001 for difference (Student's *t*‐test).

Mouse islets loaded with the green fluorescent Ca^2+^ indicator Cal‐520 were placed on top of control or GR‐cells expressing R‐GECO1 seeded on coverslips. The Cal‐520 fluorescence allowed visualization of the islet and recording of the overall islet [Ca^2+^]_i_ responses even when the image was focused on the reporter cells (Figure 2A). Depolarization of the islets with a high concentration of K^+^ (30 mM) in the presence of 3 mM glucose induced a prompt [Ca^2+^]_i_ increase in the islet cells, paralleled in GR‐cells by an initial [Ca^2+^]_i_ peak, in 16% of the cells followed by lower amplitude oscillations (Figure 2B). The GR cell [Ca^2+^]_i_ responses were inhibited by 200 nM of the SSTR2 antagonist CYN154806, demonstrating that the [Ca^2+^]_i_ signaling indeed reflected somatostatin released from the islet (Figure 2B,C). There was no effect of high K^+^ in control cells without SSTR2 (Figure 2D) or in GR cells without islets (Figure 2E). At 3 mM glucose, the K_ATP_ channel inhibitor tolbutamide evoked a less pronounced GR‐cell [Ca^2+^]_i_ response than K^+^ (Figure 2F,G). Apart from demonstrating that the GR‐cells were able to detect somatostatin release from pancreatic islets, these experiments indicate that membrane depolarization is a strong stimulus for somatostatin secretion, but that the depolarization induced by closure of K_ATP_ channels alone is not sufficient for a fulminant response.

Some GR‐cells showed [Ca^2+^]_i_ oscillations in basal medium containing 3 mM glucose when no [Ca^2+^]_i_ signaling was detected in the islet. These oscillations also reflected somatostatin secretion, since they were inhibited by CYN154806 (Figure 2H) and by 250 μM diazoxide, which hyperpolarizes δ‐cells by opening K_ATP_ channels^36^ (Figure 2I).

### 2.3 Glucose‐induced somatostatin release from mouse islets

**FIGURE 3:**
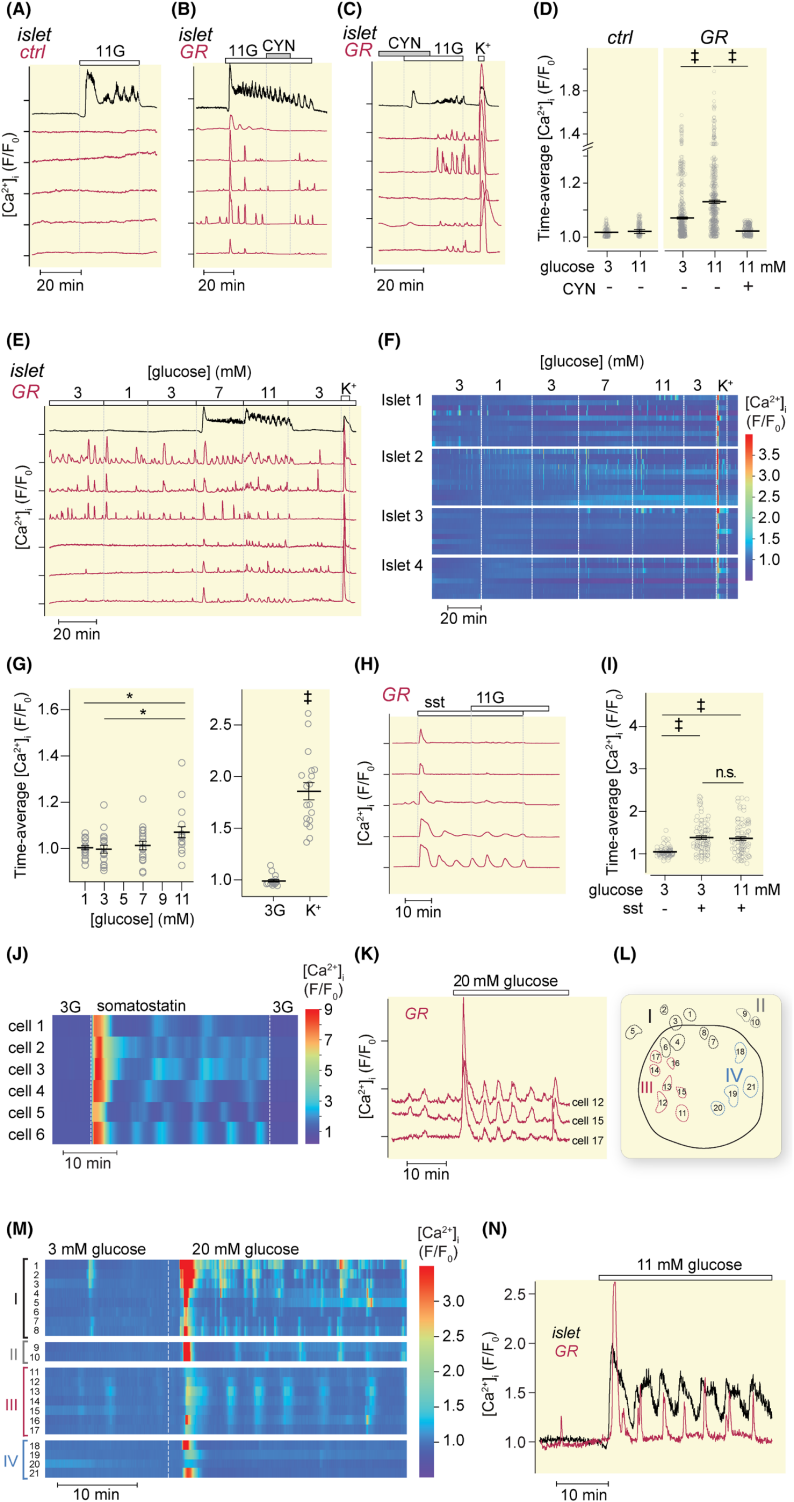
Glucose‐stimulated somatostatin release from mouse islets. (A) [Ca^2+^]_i_ recordings from mouse islets (black traces) and adjacent control cells (red traces) during elevation of the glucose concentration from 3 to 11 mM. (B, C) [Ca^2+^]_i_ recordings from mouse islets (black traces) and adjacent GR‐cells (red traces) during elevation of the glucose concentration from 3 to 11 mM and application of 200 nM CYN154806. (D) Time‐average [Ca^2+^]_i_ responses with mean ± SEM from experiments as in A and B. ‡*p* < 0.001 for difference (Student's *t*‐test). *n* = 199 cells for ctrl (three experiments) and *n* = 207 for GR‐cells (six experiments). (E) [Ca^2+^]_i_ recordings from a mouse islet and adjacent GR‐cells during exposure to different glucose concentrations and 30 mM K^+^. (F) Heatmap of [Ca^2+^]_i_ responses from GR‐cells adjacent to four different islets in experiments as in E. Each islet was associated with 8–11 GR‐cells and the response from each cell is represented as one row in the heatmap. (G) Time‐average [Ca^2+^]_i_ with mean ± SEM for the responses to glucose and high K^+^ in experiments as shown in E and F. *p < 0.05 and ‡*p* < 0.001, *t*‐test. Each point represents the average response from all cells beneath one islet. *n* = 18 islets from five experiments. (H) [Ca^2+^]_i_ recordings from GR‐cells in the absence of islets during exposure to 100 nM exogenous somatostatin followed by increase of glucose from 3 to 11 mM. (I) Time‐average [Ca^2+^]_i_ responses with mean ± SEM for experiments as in H, showing the pre‐stimulatory level, the level with sst at 3 mM glucose 1.5 min before increase of the glucose concentration, and the level with sst at 11 mM glucose (integration 10 min after increase to 11 mM). ‡*p* < 0.001 for difference (Student's *t*‐test); *n* = 86 cells from four experiments. (J) Heatmap showing oscillatory [Ca^2+^]_i_ responses in GR‐cells in the absence of islets during exposure to 3 mM glucose (3G) and 100 nM exogenous somatostatin. (K) [Ca^2+^]_i_ recordings from three closely located GR‐cells beneath an islet during exposure to an increase of glucose from 3 to 20 mM. (L) Schematic drawing of the distribution of GR‐cells beneath an islet. The corresponding [Ca^2+^]_i_ recordings are shown in panel K. Cells which tend to demonstrate similar or synchronized [Ca^2+^]_i_ responses are shown in the same color. In this experiment, four response categories were observed (I‐IV). (M) Heatmap of [Ca^2+^]_i_ responses from GR‐cells beneath an islet as shown in panel L. The cells are numbered and grouped based on response similarity as indicated in panel L. (N) [Ca^2+^]_i_ recording exemplifying synchronization of islet (black trace) and a GR‐cell (red trace) responses following increase of the glucose concentration from 3 to 11 mM.

Stimulation of islets by an increase of the glucose concentration from 3 to 11 mM triggered the characteristic tri‐phasic [Ca^2+^]_i_ response with a slight initial lowering followed by a pronounced and extended peak and subsequent elevation with oscillations, often with both fast (2–6/min) and slow (0.2–0.5/min) components (Figure 3A). Control reporter cells without SSTR2 did not show any [Ca^2+^]_i_ increases. In contrast, GR‐cells sometimes showed [Ca^2+^]_i_ oscillations at 3 mM glucose, and upon elevation to 11 mM, there was a more pronounced [Ca^2+^]_i_ increase in most cells, often followed by lower amplitude oscillations (Figure 3B). The glucose‐induced [Ca^2+^]_i_ responses in GR‐cells were inhibited by CYN154806 (Figure 3B–D), demonstrating that they were caused by somatostatin released from the islet.

The glucose sensitivity of somatostatin secretion was determined by exposing mouse islets to different concentrations in the 1–11 mM range. There were no detectable [Ca^2+^]_i_ increases in islets below 7 mM glucose (Figure 3E). However, some GR‐cells showed [Ca^2+^]_i_ activity already at 1–3 mM glucose without evident concentration dependence, whereas others responded at 3 mM, with more intense [Ca^2+^]_i_ signaling at 7 and 11 mM glucose (Figure 3E–G). Glucose did not amplify GR‐cell [Ca^2+^]_i_ signaling induced by exogenous somatostatin (Figure 3H,I), indicating that the glucose‐triggered responses detected in the presence of islets reflect stimulated somatostatin release rather than increased sensitivity of somatostatin detection. A fraction of the GR‐cells (32%) showed [Ca^2+^]_i_ oscillations in the continuous presence of 100 nM somatostatin (Figure 3H) but there was no evident synchronization of the responses between different cells (Figure 3J). In contrast, [Ca^2+^]_i_ oscillations observed in the presence of glucose‐stimulated islets were often synchronized among the reporter cells, indicating that the [Ca^2+^]_i_ kinetics reflects the temporal pattern of islet somatostatin release (Figure 3K). Closely located reporter cells often showed synchronized responses, sometimes different from those of more distantly located cells (3L, M). In some cases, slow [Ca^2+^]_i_ oscillations in islets were found to be synchronized with reporter cell [Ca^2+^]_i_ responses (Figure 3N).

### 2.4 Somatostatin secretion in response to neural, hormonal and paracrine factors

**FIGURE 4:**
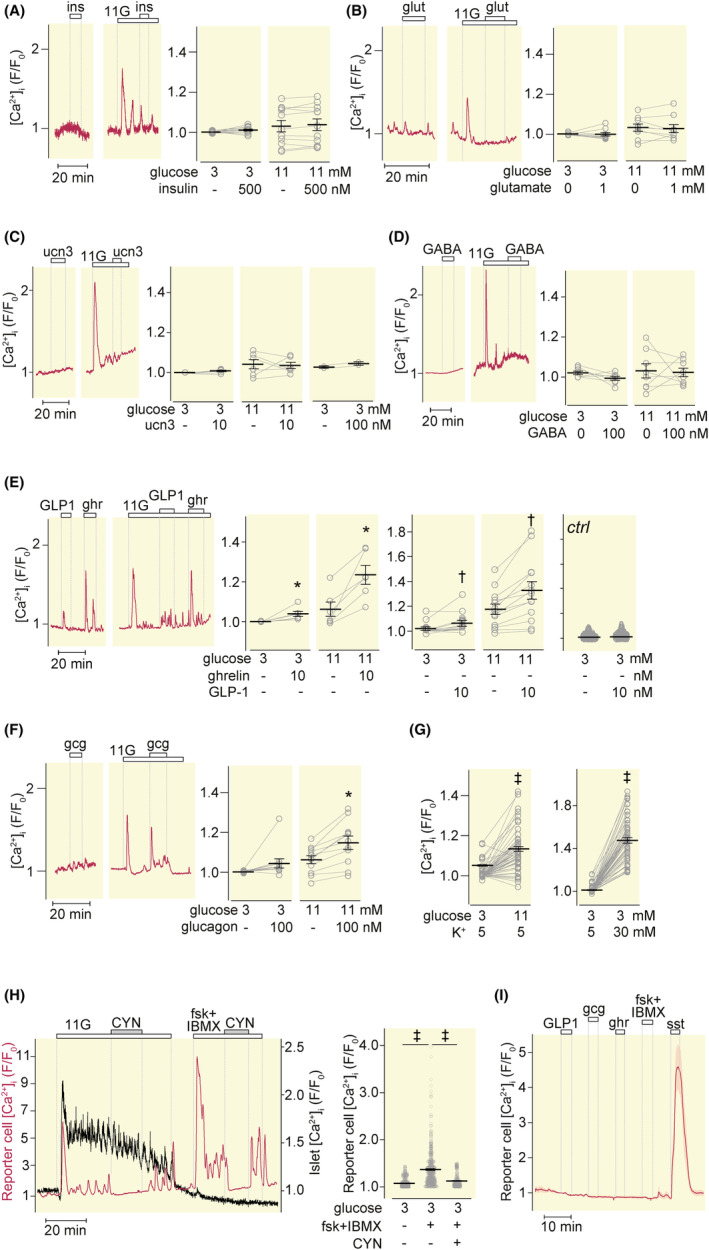
Somatostatin secretion in response to neuro‐hormonal regulators and intra‐islet paracrine factors. (A–F) [Ca^2+^]_i_ recordings from GR‐cells adjacent to mouse islets exposed to 500 nM insulin (ins, A), 1 mM glutamate (glut, B), 10 nM urocortin3 (ucn3, C), 100 nM GABA (D), 10 nM GLP‐1 and 10 nM ghrelin (E), or 100 nM glucagon (gcg, F) at 3 and 11 mM glucose. Time‐average [Ca^2+^]_i_ responses are shown in scatter plots with mean ± SEM Each point represents the average from all reporter cells beneath one islet. (A) *N* = 11 islets, 5 experiments; (B) 9 islets, 4 experiments; (C) 6 islets, 3 experiments with 10 nM and 3 experiments with 100 nM ucn3; (D) 8 islets, 3 experiments; (E) 13 islets, 4 experiments (GLP‐1), 6 islets, 3 experiments (ghrelin); (F) 11 islets, 5 experiments. *p < 0.05 and †*p* < 0.01for difference (Student's *t*‐test). (G) Time‐average [Ca^2+^]_i_ with mean ± SEM for the response to glucose elevation from 3 to 11 mM and increase of K^+^ to 30 mM. ‡*p* < 0.001 for difference (Student's *t*‐test). *N* = 52 islets from 12 experiments. (H) [Ca^2+^]_i_ recordings from a mouse islet (black trace) and GR‐cells (red trace) during elevation of glucose from 3 to 11 mM and addition of 200 nM CYN154806 and the combination of 10 μM forskolin (fsk) and 100 μM IBMX as indicated. Time‐average [Ca^2+^]_i_ responses are shown in scatter plots with mean ± SEM ‡*p* < 0.001 for difference (Student's *t*‐test); *n* = 222 cells from four experiments. (I) [Ca^2+^]_i_ recording from GR cells in the absence of islets showing mean ± SEM for the responses to 10 nM GLP‐1, 100 nM glucagon (gcg), 10 nM ghrelin, 10 μM fsk combined with 100 μM IBMX and 100 nM somatostatin (sst). Representative for three experiments, each with >40 cells.

Insulin (100 nM), glutamate (1 mM), urocortin‐3 (10 nM), and GABA (100 nM) lacked effect on [Ca^2+^]_i_ in GR‐cells with mouse islets during 5‐min exposures at both 3 and 11 mM glucose (Figure 4A–D). Urocortin‐3 lacked effect also after 10 min with 100 nM of the peptide (Figure 4C, scatter plot). In contrast, GLP‐1 (10 nM) and ghrelin (10 nM) evoked [Ca^2+^]_i_ increases at both glucose concentrations (Figure 4E). Also glucagon (100 nM) evoked [Ca^2+^]_i_ increases, although statistically significant only at 11 mM glucose (Figure 4F), indicating that these three hormones are strong positive modulators of somatostatin secretion. Nearly, all islet‐associated GR‐cells responded to high glucose and K^+^ depolarization (Figure 4G). When the cAMP concentration was increased by a combination of forskolin (10 μM) and IBMX (100 μM), there was a pronounced GR‐cell [Ca^2+^]_i_ response that was reversed by CYN154806 (Figure 4H), indicating strong stimulation of somatostatin release. None of the tested agents had effects on GR‐cell [Ca^2+^]_i_ in the absence of islets (Figure 4I).

### 2.5 Somatostatin secretion from human islets

**FIGURE 5:**
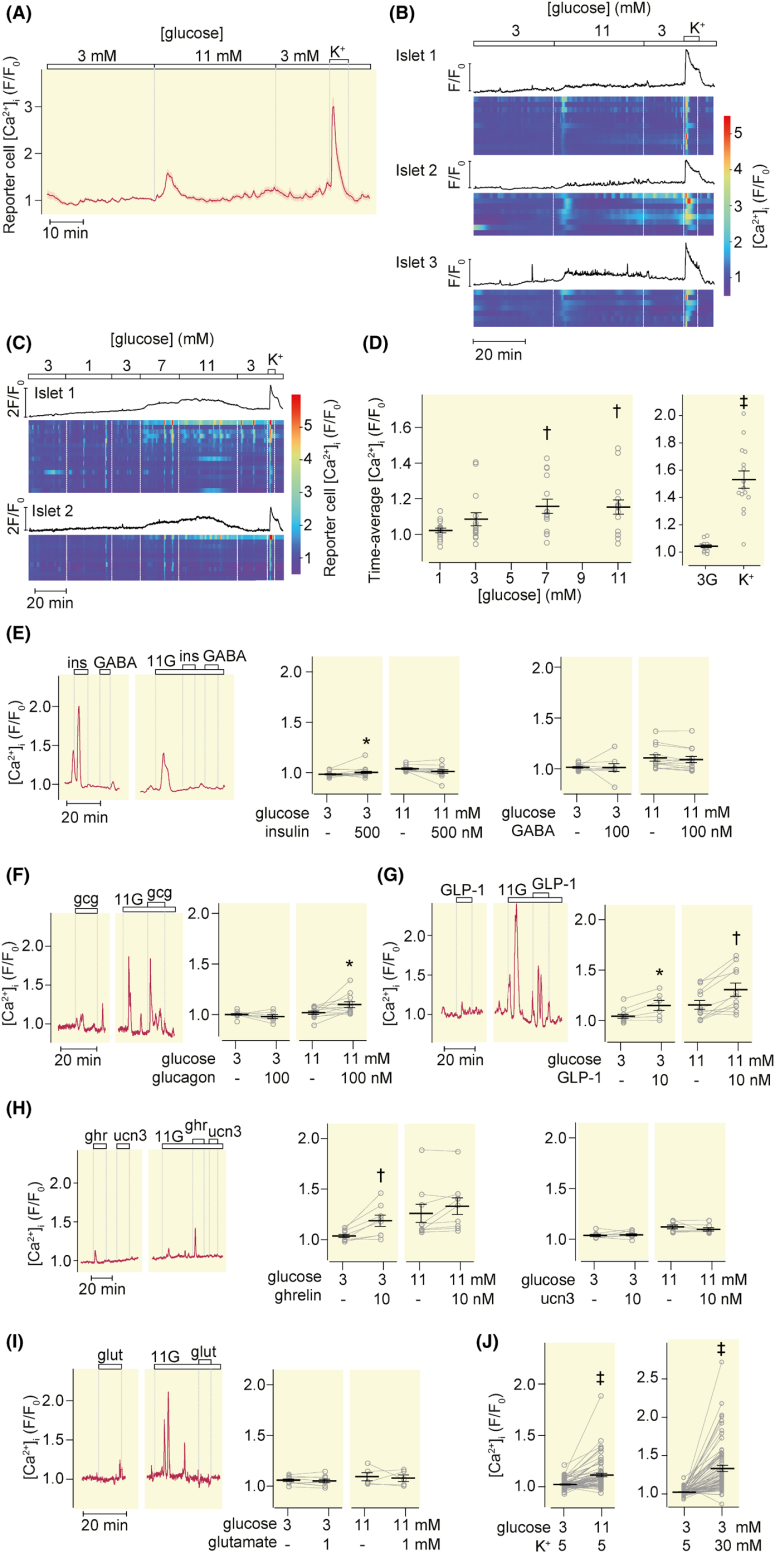
Detection of somatostatin release from healthy human donors. (A) Mean (red) ± SEM (gray) for [Ca^2+^]_i_ recorded from 27 GR‐cells adjacent to three human islets during changes of the glucose concentration from 3 to 11 mM and increase of K^+^ to 30 mM. (B) [Ca^2+^]_i_ recordings from individual human islets (black) and adjacent reporter cells (heatmaps) during stimulation with 11 mM glucose and 30 mM K^+^. Each row in the heatmap corresponds to one GR cell. (C) [Ca^2+^]_i_ recordings from human islets (black traces) and adjacent GR‐cells (heatmaps) during exposure to different glucose concentrations and 30 mM K^+^. (D) Time‐average [Ca^2+^]_i_ and mean ± SEM from experiments as in C. †*p* < 0.01; ‡*p* < 0.001 for difference (Student's *t*‐test). Each point represents the average response of all cells beneath one islet, *n* = 15 islets from five experiments. (E–I) [Ca^2+^]_i_ recordings from GR‐cells adjacent to human islets exposed to 500 nM insulin (ins) and 100 nM GABA (E), 100 nM glucagon (gcg, F), 10 nM GLP‐1 (G), 10 nM ghrelin and 10 nM urocortin‐3 (ucn3, H), 1 mM glutamate (glut, I), at both 3G and 11G. Time‐average [Ca^2+^]_i_ responses are shown in scatter plots with mean ± SEM (E) *N* = 17 islets, eight experiments (ins, 3G); 13 islets, 7 exp. (ins, 11G); 8 islets, 3 experiments (GABA, 3G), 13 islets, 5 experiments (GABA, 11G); (F) 7 islets, 3 exp. (gcg, 3G) and 12 islets, 5 exp. (gcg 11G); (G) 6 islets, 3 exp. (GLP‐1, 3G), and 11 islets, 5 exp. (GLP‐1, 11G); (H) 8 islets, 3 experiments (ghr, ucn3, 3G); 9 islets, 3 exp. (ghr, 11G), 7 islets, 3 exp. (ucn3, 11G); (I) 9 islets, 3 exp. (glu, 3G) and 5 islets, 2 experiments (glu, 11G). (J) Time‐average [Ca^2+^]_i_ with mean ± SEM for the response to glucose elevation from 3 to 11 mM and increase of K^+^ to 30 mM. ‡*p* < 0.001 for difference (Student's *t*‐test). *n* = 65 islets from 15 experiments.

Also glucose‐stimulated somatostatin secretion from human islets was detected by the Ca^2+^ responses of GR‐cells. Like for mouse islets some human islets released GR‐cell‐detected somatostatin in the presence of 3 mM glucose and increase to 11 mM always induced additional GR‐cell [Ca^2+^]_i_ activity (Figure 5A,B). Subsequent depolarization with high K^+^ caused more pronounced [Ca^2+^]_i_ increases, indicating strong stimulation of somatostatin secretion. Analysis of the glucose concentration dependence showed variability with some islets being active already at 1–3 mM, whereas others required 7 or 11 mM glucose (Figure 5C,D). The responses were inhibited by diazoxide and CYN154806, confirming that they reflected somatostatin secretion (data not shown).

Unlike with mouse islets, exposure of human islets to insulin sometimes induced GR‐cells [Ca^2+^]_i_ responses at 3 mM but not at 11 mM glucose (Figure 5E). There was also pronounced stimulation with glucagon, GLP‐1 and ghrelin (Figure 5F–H), but no effects of GABA, urocortin‐3 or glutamate (Figure 5E,H,I). All islets induced GR‐cell responses to 11 mM glucose and 30 mM K^+^ (Figure 5J).

### 2.6 Excessive glucose‐ and depolarization‐stimulated somatostatin release from human type 2 diabetic islets

**FIGURE 6:**
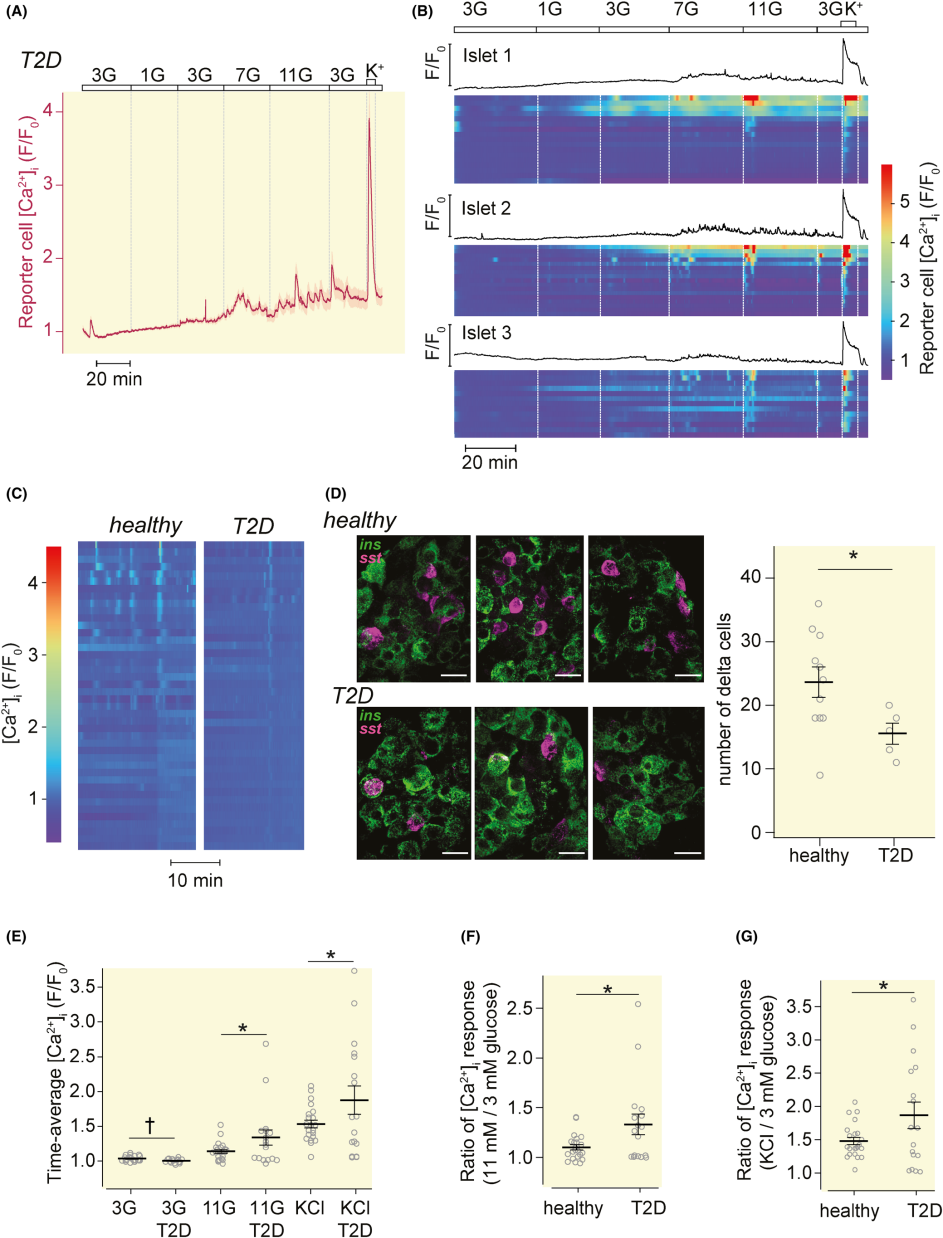
Somatostatin release from human type 2 diabetes islets. (A) Mean (red) ± SEM (gray) for [Ca^2+^]_i_ recorded from 50 GR‐cells adjacent to three human islets during exposure different glucose concentrations and increase of K^+^ to 30 mM. (B) [Ca^2+^]_i_ recordings from individual islets (black) and adjacent reporter cells (heatmaps) during stimulation with different glucose concentrations and 30 mM K^+^. Each row in a heatmap corresponds to one GR cell. (C) Heatmaps of [Ca^2+^]_i_ responses from GR‐cells adjacent to islets from healthy and type 2 diabetes donors exposed to 3 mM glucose showing higher activity in islets from healthy donors. (D) Confocal images from healthy or T2D human islets immunostained for insulin (green) and somatostatin (magenta). Scale bar, 10 μm. The total number of somatostatin‐positive cells per islet is shown in scatter plots with mean ± SEM *p < 0.05 for difference (Student's *t*‐test). *n* = 11 islets from 3 healthy donors and 5 islets from two T2D donors. (E) Time‐average [Ca^2+^]_i_ at 3 and 11 mM glucose (3G, 11G) and at 3 mM glucose with 30 mM K^+^ for healthy and type 2 diabetes islets are shown in scatter plots with mean ± SEM *p < 0.05 and †*p* < 0.01 (Student's *t*‐test). *n* = 22 islets from five healthy donors and *n* = 17 islets from four T2D donors. (F, G) [Ca^2+^]_i_ responses expressed as the ratio of 11 vs. 3 mM glucose (F) and high K^+^ vs. 3 mM glucose (G). Scatter plots with mean ± SEM *p < 0.05 and ‡*p* < 0.001 for difference (Student's *t*‐test). *n* = 22 islets from 5 healthy donors (nine experiments) and *n* = 17 islets from four T2D donors (six experiments).

Islets obtained from four human type 2 diabetic donors induced GR‐cell [Ca^2+^]_i_ responses when the glucose concentration was increased from 3 to 7 or 11 mM and after depolarization with high K^+^ (Figure 6A,B). At 3 mM glucose, there were somewhat less GR‐cell [Ca^2+^]_i_ activity with type 2 diabetic compared with normal islets (Figure 6C). Immunostaining for somatostatin followed by quantification of cell numbers with confocal microscopy showed a lower number of δ‐cells in diabetic islets (Figure 6D). The GR‐cell [Ca^2+^]_i_ responses when islets were exposed to 11 mM glucose or high K^+^ were nevertheless more pronounced with type 2 diabetic than normal islets (Figure 6E). The difference was significant also when comparing the stimulation index (11 vs. 3 mM and high vs. normal K^+^) for healthy and type 2 diabetes islets (Figure 6F,G). Together, these results indicate that type 2 diabetes is associated with reduced somatostatin secretion at 3 mM glucose, perhaps because of a reduced number of δ‐cells, but an increased response to high glucose and depolarization.

## 3 DISCUSSION

Detection of somatostatin release is critical for understanding the role of this paracrine factor in islet physiology. The limited sensitivity of commercially available immunoassays makes it necessary to collect samples from large groups (10s to 100s) of islets which restricts the possibilities to obtain kinetic information. It is also difficult to simultaneously record islet signaling. This study describes the generation of a reporter cell assay for somatostatin based on [Ca^2+^]_i_ recordings in cells with heterologous expression of SSTR2. The assay is inexpensive, straightforward and allows real‐time monitoring of somatostatin secretion kinetics from individual islets while at the same time recording [Ca^2+^]_i_ in islet cells. A recent study utilized SSTR5 receptors engineered to contain a fluorescent protein to detect somatostatin release from isolated islets.^37^ This approach provides information on somatostatin release with high spatial resolution but seems less efficient for determining total release from the islets. Reporter cell assays using [Ca^2+^]_i_ as readout obviously only provide semi‐quantitative information, and there is also a risk for unspecific responses. The latter problem can be handled with appropriate control experiments and we here demonstrate the utility of the assay for determining somatostatin secretion from both mouse and human islets.

Among different somatostatin receptors, we chose SSTR2, a subtype expressed in α‐cells,^8^, ^13^ to achieve sensitivity relevant for intra‐islet paracrine regulation of glucagon secretion. SSTR2 mainly couples via Gαi, and cAMP could therefore have been selected as readout, but since cAMP concentrations usually are low in unstimulated cells, reductions in cAMP generation would be difficult to detect. We found that SSTR2 in HeLa cells naturally coupled to Gq and phospholipase C‐dependent Ca^2+^ signaling, but phospholipase C activation was improved and sensitivity of somatostatin detection increased sixfold when co‐expressing the Gq family member Gα15, which is known to couple a wide range of receptors to phospholipase C,^38^ SSTR2 has been reported to internalize and desensitize in response to high agonist concentrations^39^ but we did not observe any loss of reporter cell responsivity in the presence of islets and many experiments were ended by stimulation of somatostatin secretion with depolarization or by directly adding somatostatin to the reporter cells.

As expected, glucose stimulated somatostatin secretion, but a large variability was noted. Some islets showed a relatively high secretion already at 1 mM glucose, with increasing concentrations inducing transiently elevated secretion as if δ‐cells sense the change rather than the absolute glucose level. This observation is consistent with [Ca^2+^]_i_ measurements in δ‐cells identified by transgenic expression of fluorescent proteins in mouse islets.^40^ Other islets showed a threshold for glucose stimulation of somatostatin secretion at ~7 mM. Some variability in reporter cell responses likely reflect differences in SSTR2 expression and local differences in somatostatin concentration depending on the location of the cells in relation to δ‐cells in the islet. Such variation should be reduced when averaging the response from a number of cells beneath an islet. In view of the stimulatory effect of glucagon on somatostatin secretion,^41^ variability in reporter cell responses between islets at low glucose may also reflect differences in the islet content of either α‐ or δ‐cells, or both. Reporter cells frequently showed oscillatory [Ca^2+^]_i_ responses. We conclude that such oscillations synchronized among closely located reporter cells reflect the kinetics of somatostatin release since no synchronization was observed when the reporter cells were exposed to constant somatostatin in the absence of islets.

In line with previous studies using immunoassays and single‐cell capacitance measurements, demonstrating a role for K_ATP_ channels in glucose regulation of somatostatin secretion,^7^, ^36^, ^42^ we found that somatostatin release was inhibited by diazoxide and stimulated by tolbutamide. The tolbutamide response was weaker than that of both depolarization with 30 mM K^+^ and 11 mM glucose, indicating that closure of K_ATP_ channels is not sufficient for a fulminant secretion response and that additional mechanisms contribute to the effect of glucose.

A strength with this reporter cell assay is the possibility to simultaneously detect [Ca^2+^]_i_ in the islet cells. The present approach with widefield microscopy recordings did not allow recordings from individual cells, but the integrated islet response showed [Ca^2+^]_i_ signaling patterns typical for β‐cells with both slow oscillations and overlayed faster ones.^43^, ^44^, ^45^, ^46^ The slow islet [Ca^2+^]_i_ oscillations were sometimes well synchronized with the reporter cell responses, suggesting that there are pulses of somatostatin secretion in phase with β‐cell insulin secretion. Such synchronization has previously been demonstrated in the perfused rat pancreas^47^ and in perifused mouse^27^ and human islets.^26^ Also [Ca^2+^]_i_ oscillations have been found to be synchronized between β‐ and δ‐cells.^40^ Paracrine mechanisms can contribute to such synchronization, but there is also evidence for electrical coupling between β‐ and δ‐cells.^28^

Cyclic AMP strongly potentiated somatostatin secretion even at low glucose concentrations. Previous studies have suggested that cAMP induces Ca^2+^‐induced Ca^2+^ release via Epac‐dependent activation of ryanodine receptors.^48^ The mechanism underlying the cAMP response was not investigated here but the potent effect of cAMP provides an explanation for the stimulatory action of glucagon and GLP‐1 on somatostatin secretion.

Human islets were similar to mouse islets in terms of glucose responsiveness, although the concentration dependence was not particularly pronounced. In contrast to previous reports, we did not detect any effect of glutamate,^25^ GABA^49^ or urocortin‐3.^24^ The reason for the discrepancy is not clear. Perhaps the responses are below the detection limit for the reporter cell assay, or the receptors were already activated by high endogenous concentrations of the neurotransmitters.

The most robust physiological stimulator of somatostatin secretion was ghrelin, a hormone/paracrine factor that within islets is produced by a minor population of ε‐cells^50^ but immunoreactivity has been reported also from α‐^51^ and β‐cells.^52^ A stimulatory action of ghrelin on somatostatin secretion is in line with previous reports^22^, ^23^ but the physiological relevance of the effect remains unclear. Factors released from α‐ and β‐cells appears to stimulate somatostatin release, which in turn acts as a brake by feedback‐inhibiting secretion of insulin and glucagon. In human islets, insulin stimulated somatostatin secretion at low glucose. Lack of effect at high glucose may reflect the already high insulin concentrations. It is possible that the effect of insulin contributes to the stimulatory effect of glucose on somatostatin release. Added glucagon and GLP‐1 mainly amplified secretion at high glucose when endogenous glucagon levels are low. On the other hand, by increasing δ‐cell cAMP, native glucagon may be able to drive somatostatin secretion at low glucose concentrations.

Islets from type 2 diabetic donors showed a reduced number of δ‐cells, which is in line with a recent report.^53^ It is also consistent with the observation that the total pancreatic somatostatin content is reduced in type 2 diabetic subjects.^54^ One study,^55^ but not others,^56^, ^57^ described a reduced volume density of δ‐cells in diabetic patients.^56^, ^57^ It is therefore surprising that the responses to glucose and K^+^ depolarization were significantly larger in the diabetic islets. This indicates that the remaining δ‐cells hypersecrete somatostatin. Previous studies of somatostatin secretion in type 2 diabetes models have yielded divergent results. Somatostatin secretion from genetically obese^24^ or high fat‐fed mouse islets^58^ has been reported to be reduced, mirroring the effects of long‐term exposure of islets to free fatty acids.^59^ In contrast, somatostatin secretion was enhanced, at least at low glucose concentrations, in a non‐obese diabetic mouse model^60^ and in human islets from type 2 diabetic donors.^60^ Such increased responsiveness was supported by the present observations and may be an inherent δ‐cell phenomenon, but might potentially also reflect increased glucagon secretion at high glucose concentrations. Interestingly, α‐cells from type 2 diabetic patients show reduced surface expression of somatostatin receptors and thereby resistance to somatostatin,^61^ but it is difficult to know if that is a cause or an effect of increased somatostatin secretion.

## 4 MATERIALS AND METHODS

### 4.1 Materials

All cell culture reagents were from Life technologies (Carlsbad, CA, USA). SSTR2 antagonist CYN154804 and GLP‐1 were from Tocris Bioscience (Bristol, UK). Ghrelin and urocortin 3 were from Bachem (Bubendorf, Switzerland), whereas insulin, glucagon, somatostatin‐14, forskolin, 3‐isobutyl‐methylxantine (IBMX), GABA, glutamate, and salts for the superfusion buffer were from Sigma‐Aldrich (St Louis, MO, USA).

### 4.2 Plasmid construction

cDNA for Gα15 and SSTR2 was obtained from UMR cDNA Resource Center (Bloomsburg, PA, USA) and an internal ribosome entry site (IRES) was isolated from the pTRE3G‐IRES plasmid (Clontech, Göteborg, Sweden). Plasmids were generated to express SSTR2 alone or together with Gα15. Restriction sites *Mlu*I (N‐terminal) and *Xba*I (C‐terminal) were used in primers for SSTR2 (forward 5′‐CCCACGCGTACCATGGACATGGCGGATGAGCCA‐3′ and reverse 5′‐CCGTCTAGATCAGATACTGGTTTGGAGGTCT‐3′). Restriction sites *Not*I (N‐terminal) and *Sal*I (C‐terminal) were used in primers for Gα15 (forward 5′‐CCCGCGGCCGCACCATGGCCCGCTCGCTGACCT‐3′ and reverse 5′‐CCGGTCGAC‐TCACAGCAGGTTGATCTCGT‐3′). The Gα15, SSTR2, and IRES fragments were subsequently ligated into the vector pSF‐CMV‐PGK‐Puro (Sigma‐Aldrich), which was used for cell transfection. The final plasmid construct was verified by sequencing.

### 4.3 HeLa cell culture and transfection

HeLa cells were cultured in DMEM medium supplemented with 2 mM L‐glutamine, 100 U/mL penicillin, 100 μg/mL streptomycin and 10% fetal bovine serum, at 37°C and 5% CO_2_. The cells were transfected with either of the two SSTR2 constructs and the Ca^2+^ indicator R‐GECO1 during seeding onto poly‐D‐lysine‐coated, 25‐mm coverslips in 35‐mm petri dishes. A mixture of 100 μL OptiMEM medium with 50 000 HeLa cells, 0.2 μg plasmid DNA and 0.5 μL Lipofectamine 2000 was added to each coverslip. After 3 hours, 2 mL DMEM medium was added to each dish and the cells were cultured for 2–3 days.

### 4.4 Islet isolation and culture

Pancreatic islets were collagenase‐isolated from C57Bl6J mice (Scanbur, Sollentuna, Sweden) and cultured in RPMI 1640 medium with 11 mM glucose, 2 mM L‐glutamine, 100 U/mL penicillin, 100 μg/mL streptomycin and 10% fetal bovine serum at 37°C in a 5% CO_2_ humidified air atmosphere for 2–3 days. All animal experimental procedures were approved by the Uppsala animal ethics committee. The effects of glucose were investigated with islets isolated from animals of both sexes, whereas all other experiments were carried out on islets from female mice. No influence of animal sex on the results was observed.

Human islets from eight donors without and four donors with type 2 diabetes (T2D) were provided by the Nordic Network for Clinical Islet Transplantation. Donor characteristics are presented in Table 1. Experimental handling was approved by Uppsala human ethics committee. After delivery to the laboratory, the islets were maintained in CMRL 1066 medium with 5.5 mM glucose, 2 mM glutamine, 100 U/mL penicillin, 100 μg/mL streptomycin and 10% fetal bovine serum for 3–5 days at 37°C and 5% CO_2_.

**TABLE 1:** Human islet donor characteristics.

Islet preparation	Diabetes	Sex	Age, years	BMI, kg/m^2^	HbA1c, mmol/mol	Related experiments
H2599	‐	Female	54	18.0	36	Figure 5I,J
H2606	‐	Male	46	19.4	34	Figure 5E–H,J
H2608	‐	Male	49	27.5	33	Figure 5E–H,J
H2610	‐	Male	62	22.2	36	Figures 5C,D and 6E–G
H2613	‐	Male	65	22.3	38	Figures 5D,E,I,J and 6D–G
H2614	‐	Male	69	27.1	Unknown	Figures 5D,E,J and 6D–G
H2627	‐	Male	61	27.4	41	Figures 5A,B,D–G,J and 6D–G
H2636	‐	Female	48	28.8	43	Figures 5D,E,G–J and 6E–G
H2601	T2D	Male	80	31.1	Unknown	Figure 6E–G
H2611	T2D	Male	51	Unknown	58	Figure 6E–G
H2618	T2D	Male	80	27.7	45	Figure 6A–G
H2622	T2D	Female	86	24.4	38	Figure 6D–G

### 4.5 Imaging of the cytoplasmic Ca^2+^ concentration ([Ca^2+^]_i_) in islets and reporter cells

[Ca^2+^]_i_ imaging experiments were performed with a basal buffer containing 125 mM NaCl, 4.8 mM KCl, 1.3 mM CaCl_2_, 1.2 mM MgCl_2_, 1 mg/mL BSA and 25 mM HEPES (pH adjusted to 7.40 with NaOH). Unless otherwise stated, the glucose concentration was 3 mM. Islets were loaded with 1 μM fluorescent Ca^2+^ dye Cal‐520 acetoxymethyl ester (AAT Bioquest, Sunnyvale, CA, USA) by 30–60 min incubation at 37°C immediately before imaging. HeLa cells were transfected with the genetically encoded Ca^2+^ sensor R‐GECO1 and cultured 2–3 days before being transferred to experimental buffer and preincubated 30–60 min at 37°C before imaging. The coverslips with HeLa cells were used as bottoms of a 50‐μL superfusion chamber and the islets were subsequently applied on top of the HeLa cells. The chamber was mounted on the stage of an Eclipse TE2000 microscope (Nikon) with a 40× objective (NA 1.3). An LED light source (LedHUB, Omicron Laserage Laserprodukte GmbH, Rodgau, Germany) provided excitation light at 488 nm/10 nm half bandwidth (Cal‐520) and 561/4 nm (for R‐GECO1) reflected onto the sample by a zt405/488/561/640rpc‐UF2 dichroic mirror (Chroma Technology Group). Emission was measured at 530/30 and 609/62 nm, respectively, using an EMCCD camera (DU‐897 Andor Technology, Belfast, Northern Ireland, UK). Images were acquired every 3 s using MetaFluor software (Molecular Devices, Downingtown, PA, USA), which was also employed for analysis. Igor Pro (Wavemetrics, Lake Oswego, OR) was used for data analysis and together with Illustrator (Adobe Systems, San Jose, CA) for figure preparation. Fluorescence intensities (F) from single cells or islets were normalized to the initial pre‐stimulatory intensity (F_0_) after background subtraction and expressed as (F/F_0_). Unless otherwise stated, reporter cell responses were quantified by calculating the time‐averaged area‐under‐curve for the entire stimulation period. For the data in Figures 4A–G and 5E–J, integration was restricted to the first 3 min of each condition. In experiments with islets stimulated with glucose, hormones and neurotransmitters, high K^+^ was used as a positive control, and only reporter cells that responded to the islet depolarization were included in the analyses.

### 4.6 Immunofluorescence staining and confocal imaging

Islets were collected after [Ca^2+^]_i_ imaging and washed with PBS, followed by fixation with 4% paraformaldehyde for 20 min and permeabilization with 0.2% Triton X‐100 for 5 min. Blocking was performed with 2% BSA for 1 h at room temperature before adding the primary antibodies against somatostatin (A0566, host: rabbit, 1:500) and insulin (A0564, host: guinea pig, 1:500; both from Dako, Sundbyberg, Sweden). After 2 h incubation at room temperature, the islets were washed with PBS and incubated for 1 h with the secondary antibodies anti‐rabbit 647 nm for somatostatin (A32795; 1:500) and anti‐guinea pig 488 nm for insulin (A11073; 1:500; both from Invitrogen, Göteborg, Sweden). The islets were subsequently washed with PBS and mounted on a glass slide with a few drops of 1% agar as embedding medium.

Images of immunolabeled islets were acquired with an Eclipse Ti2 microscope (Nikon) equipped with a Yokogawa CSU‐X1A spinning disk confocal system (Visitron Systems GmbH) and a 100x, 1.49‐NA oil immersion objective. A 491 nm diode‐pumped solid‐state laser and a 640‐nm diode laser (Cobolt AB, Solna, Sweden) were used for excitation and fluorescence was selected with 530/50 nm and 650 nm long‐pass filters (Semrock) and recorded with an EMCCD camera (DU‐888, Andor Technology).

## 5 CONCLUSIONS

We have developed a reporter cell assay for semi‐quantitative monitoring of somatostatin secretion from pancreatic islets. The sensitivity allows real‐time detection of somatostatin from individual islets. We confirm previous observations that secretion is regulated by glucose and highly sensitive to cAMP. We also found that ghrelin is a robust stimulator of somatostatin secretion but in contrast to the general view, we did not find support for regulation by glutamate, GABA or urocortin‐3. Diabetic islets show increased secretion of somatostatin, and this change may lead to altered secretion of the other islet hormones.

## AUTHOR CONTRIBUTIONS

**Mingyu Yang:** Methodology; investigation; formal analysis; writing – original draft. **Kousik Mandal:** Software; visualization. **Moa Södergren:** Investigation. **Özge Dumral:** Investigation. **Lena Winroth:** Investigation; methodology. **Anders Tengholm:** Conceptualization; supervision; writing – review and editing.

## FUNDING INFORMATION

This work was supported by grants from the Swedish Research Council (2021‐02081), Swedish Diabetes Foundation, Diabetes Wellness Foundation, Family Ernfors Foundation, Novo Nordisk Foundation (NNF20OC0064000), the Leona M and Harry B Helmsley Charitable Trust, the Swedish Child Diabetes Foundation and the Swedish national strategic grant initiative EXODIAB (Excellence of diabetes research in Sweden). Human islets were generously provided by the Nordic Network for Clinical Islet Transplantation supported by EXODIAB.

## CONFLICT OF INTEREST STATEMENT

The authors have no competing interests to declare.

## Data Availability

Data will be made available on request.
